# Influence of Gate Geometry on the Characteristics of AlGaN/GaN Nanochannel HEMTs for High-Linearity Applications

**DOI:** 10.3390/mi14081513

**Published:** 2023-07-28

**Authors:** Meng Zhang, Yilin Chen, Siyin Guo, Hao Lu, Qing Zhu, Minhan Mi, Mei Wu, Bin Hou, Ling Yang, Xiaohua Ma, Yue Hao

**Affiliations:** 1 School of Microelectronics, Xidian University, Xi’an 710071, China; 2 Key Laboratory of Wide Band-Gap Semiconductor Materials and Devices, School of Microelectronics, Xidian University, Xi’an 710071, China

**Keywords:** GaN, high electron mobility transistors, nanochannel, tri-gate, dual-gate

## Abstract

In this study, AlGaN/GaN nanochannel high-electron-mobility transistors (HEMTs) with tri-gate (TGN-devices) and dual-gate (DGN-devices) structures were fabricated and investigated. It was found that the peak value of the transconductance (G_m_), current gain cut-off frequency (f_T_) and power gain cut-off frequency (f_max_) of the TGN-devices were larger than that of the DGN-devices because of the enhanced gate control from the top gate. Although the TGN-devices and DGN-devices demonstrated flattened transconductance, f_T_ and f_max_ profiles, the first and second transconductance derivatives of the DGN-devices were lower than those of the TGN-devices, implying an improvement in linearity. With the nanochannel width decreased, the peak value of the transconductance and the first and second transconductance derivatives increased, implying the predominant influence of sidewall gate capacitance on the transconductance and linearity. The comparison of gate capacitance for the TGN-devices and DGN-devices revealed that the gate capacitance of the tri-gate structure was not simply a linear superposition of the top planar gate capacitance and sidewall gate capacitance of the dual-gate structure, which could be attributed to the difference in the depletion region shape for tri-gate and dual-gate structures.

## 1. Introduction

Gallium nitride (GaN)-based high-electron-mobility transistors (HEMTs) have great potential for high-frequency and high-power applications because of the advantages of heterojunction materials, including their high breakdown electric field, high two-dimensional electron gas (2DEG) sheet density and high electron mobility [1,2,3,4], leading to potential RF applications, including remote sensing, radar and wireless communication [5,6]. Except for the superiority of GaN-based HEMTs mentioned above, linearity is also a key characteristic for RF applications, especially for wireless communication. However, the transconductance nonlinearity, which is defined as the reduction of transconductance and f_T_ at a high drain current density level in GaN-based HEMTs, can limit the device linearity, leading to distortion of the signal [7,8]. Several structures can be used to alleviate this, such as graded polarization field effect transistors [9], double-channel heterojunctions [10], coupling-channel structures [11], N-polar HEMTs [12] and nanochannel structures [13]. Aside from these structures, nanochannel structures have attracted more attention because of the additional improvement in the electron velocity [14], self-heating effect [15], subthreshold swing [16], breakdown voltage [17] and so on. Moreover, a nanochannel structure can modulate the threshold voltage by varying the nanochannel width [18], which is also helpful for the improvement in linearity via threshold voltage synthesis and transconductance compensation [8,19,20].

For a nanochannel structure, there are two different gate structures, namely, a tri-gate structure [21,22] and dual-gate structure [23,24,25,26], which demonstrate the potential for the improvement in device linearity. Compared with tri-gate GaN HEMTs with multiple channels [27], dual-gate GaN HEMTs for multi-channel epitaxial design can achieve flattened transconductance, and thus, improve the device linearity and saturated current density simultaneously [25,26]. However, the characteristic difference between tri-gate and dual-gate structures has rarely been compared simultaneously. Moreover, the influence of source resistance nonlinearity on the linearity of GaN-based nanochannel HEMTs with tri-gate and dual-gate structures has been developed, but the influence of the capacitance from the sidewall gate was less mentioned.

In this work, AlGaN/GaN nanochannel HEMTs with tri-gate (TGN-devices) and dual-gate structures (DGN-devices) were fabricated and investigated. The DC characteristics and small-signal characteristics were compared and analyzed. The TGN-devices demonstrated a higher peak value of transconductance and cut-off frequency than that of the DGN-devices, but the DGN-devices presented lower second transconductance derivatives, implying better linearity. The gate capacitances of the TGN-devices and DGN-devices were compared and the influence of the capacitance from the sidewall gate is discussed.

## 2. Device Fabrication

**Figure 1:**
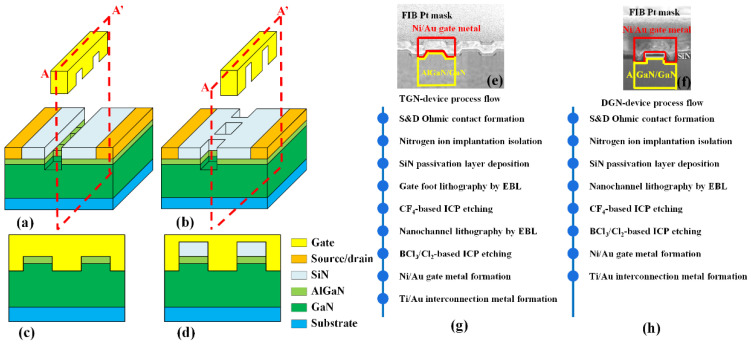
Schematic of a (**a**) TGN-device and (**b**) DGN-device; cross-section view of a (**c**) TGN-device and (**d**) DGN-device along the AA’ position; cross-section FIB-SEM photo of a (**e**) TGN-device and (**f**) DGN-device along the AA’ position; fabrication process flow of a (**g**) TGN-device and (**h**) DGN-device.

The schematics of the TGN-devices and DGN-devices investigated in this manuscript are shown in Figure 1. The TGN-devices and DGN-devices were fabricated on the same wafer. The epitaxial heterostructure consisted of a 100 nm AlN nuclear layer, a 2 μm GaN buffer layer, a 1 nm AlN interlayer and a 20 nm AlGaN barrier layer with an aluminum composition of 23% from bottom to top, grown on a sapphire substrate. A sheet density of 369 Ω/□, a two-dimensional electron gas (2DEG) of 9.1 × 10^12^ cm^−2^ and 2DEG mobility of 1860 cm^2^/V∙s was obtained via Hall measurements at room temperature. The fabrication process of devices started with the formation of an ohmic contact on the source and drain using conventional Ti/Al/Ni/Au (20/160/55/45 nm) metal stack evaporation, followed by rapid annealing at 850 °C for 50 s in ambient N_2_. After the device’s electrical isolation was realized via nitrogen implantation, an ohmic contact resistance of 0.5 Ω∙mm was verified using a transmission line measurement (TLM). A 120 nm SiN layer was deposited for surface passivation via plasma-enhanced chemical vapor deposition (PECVD). For the TGN-devices, as shown in Figure 1a,c, the nanochannel was surrounded and contacted from three directions by the Ni/Au gate metal, including the top and two sidewalls of the nanochannel. Figure 1e shows the cross-section FIB-SEM photo of a TGN-device. Figure 1g shows the fabrication process flow of a TGN-device. The gate foot defined a gate length (L_g_) of 0.2 μm using electron beam lithography (EBL) and CF_4_-based inductively coupled plasma (ICP) etching to remove SiN on the gate region. Then, a nanochannel with a width (W_fin_) of 150, 200 or 250 nm was defined using EBL, followed by BCl_3_/Cl_2_-based ICP etching. As shown in Figure 1b,d, for the DGN-devices, although the nanochannel was surrounded from three directions by the Ni/Au gate metal, the nanochannel was contacted by the Ni/Au gate on the two sidewalls and there was a 120 nm thick SiN passivation dielectric between the top of the nanochannel and the Ni/Au gate. The PECVD SiN was amorphous and would not exert an influence on the polarization charges. Figure 1f shows the cross-section FIB-SEM photo of a DGN-device. Figure 1h shows the fabrication process flow of a DGN-device. The nanochannel width of the DGN-devices was equal to that of the TGN-devices and was directly realized via EBL, CF_4_-based ICP etching and BCl_3_/Cl_2_-based ICP etching in sequence. The length of the nanochannel was equal to the gate length for both structures. The nanochannel width (W_fin_) and the trench (W_trench_) region were equal for both structures. The gate electrode (Ni/Au) with a gate cap length of 1.0 μm was formed via physical vapor deposition and a lift-off process. Finally, the interconnection via Ti/Au metalization for the device test was achieved. In this study, all devices had the same gate width of 100 μm, source–drain distance of 4 μm and source–gate distance of 0.9 μm.

## 3. Results and Discussion

**Figure 2:**
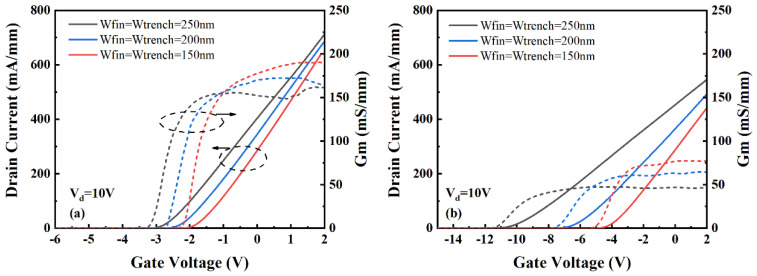
The transfer characteristics of the (**a**) TGN-devices and (**b**) DGN-devices. The nanochannel widths were 150, 200 and 250 nm, respectively. The straight and dotted lines standed for drain current density and tranconductance, respectively.

**Figure 3:**
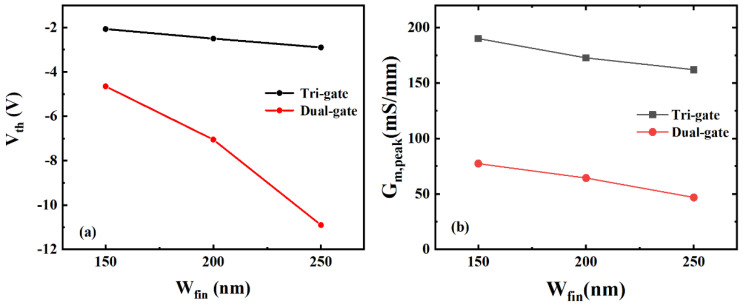
The dependence of (**a**) the threshold voltage (V_th_) and (**b**) peak transconductance (G_m,peak_) dependence on the nanochannel width (W_fin_) for the TGN-devices and DGN-devices.

The transfer characteristics of the TGN-devices and DGN-devices are shown in Figure 2. The drain was biased at 10 V. The nanochannel widths were 150, 200 and 250 nm. The DC performance was normalized to the actual gate width. As presented in Figure 2, with the reduction in the nanochannel width, the threshold voltage (V_th_) and the peak value of transconductance (G_m,peak_) for both the TGN-devices and DGN-devices increased, which was mainly attributed to the enhanced electrostatic gate control from the sidewall gate and the reduction in the polarization charge density induced by the tensile strain relaxation [13,28]. Both the TGN-devices and DGN-devices demonstrated flattened transconductance. Figure 3 shows the V_th_ and G_m,peak_ dependence on the nanochannel width (W_fin_) for the TGN-devices and DGN-devices. As shown in Figure 3a, the gradients of the V_th_–W_fin_ curves for the TGN-devices and DGN-devices were different. As W_fin_ increased, the V_th_ of the TGN-devices decreased slowly, while that of the DGN-devices decreased rapidly. For the TGN-devices, it was implied that the influence of the sidewall gates on V_th_ was ancillary and that of the top gate was predominant, contributing to the reduced slope of the V_th_–W_fin_ relationship. As W_fin_ increased continuously, the V_th_ of the TGN-devices approximated the V_th_ of conventional planar GaN-based HEMTs and was limited [18]. However, for the DGN-devices, the influence of the sidewall gates on V_th_ dominated, indicating the strong dependence of V_th_ on W_fin_. Moreover, because of the lack of control from the top gate in the dual-gate structure, as W_fin_ increased continuously, the threshold voltage of the DGN-devices decreased continuously without restriction. When further decreasing W_fin_ to less than 100 nm, the V_th_ of the TGN-devices and DGN-devices could be approximated [29], and the normally off TGN-devices and DGN devices could be realized. As shown in Figure 3b, the gradients of the G_m,peak_–W_fin_ curves for the tri-gate structure and dual-gate structure were similar, indicating the similar electrostatic charge control effect from the sidewall gates. For the TGN-devices and DGN-devices with the same W_fin_, the G_m,peak_ of the TGN-devices was approximately 110 mS/mm larger than that of the DGN-devices due to the increase in the capacitance from the top gate in the tri-gate structure.

**Figure 4:**
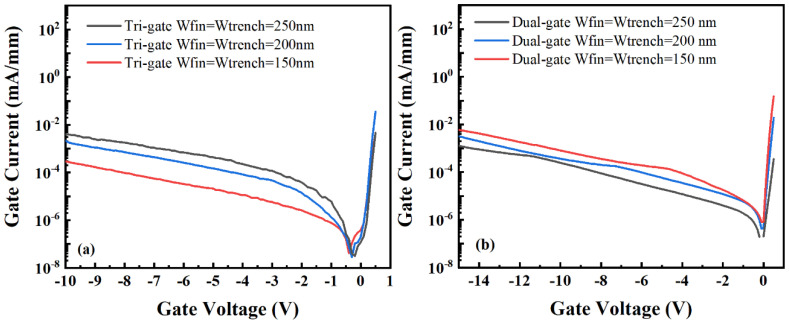
Gate current versus gate voltage for the (**a**) TGN-devices and (**b**) DGN-devices.

**Figure 5:**
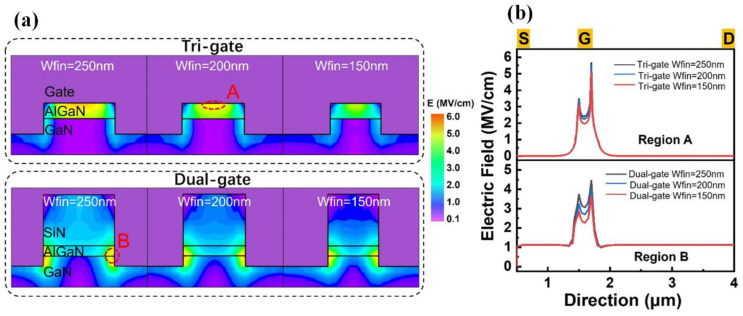
(**a**) The transverse distribution of the electric field and (**b**) the electric field distribution along the nanochannel length direction of the TGN-devices and DGN-devices with W_fin_ values of 150, 200 and 250 nm.

Figure 4 presents the gate currents for the TGN-devices and DGN-devices. As can be seen in Figure 4, for the TGN-devices, with the increase in W_fin_, the reverse gate leakage current increased. However, for the dual-gate structure, with the increase in W_fin_, the reverse gate leakage current was reduced. This could be attributed to the different electric field distribution dependences on W_fin_ for the tri-gate and dual-gate structures. The simulated transverse distributions of the electric field and the electric field distribution (parallel to the nanochannel length direction) of the TGN-devices and DGN-devices with W_fin_ values of 150, 200 and 250 nm are shown in Figure 5, where the simulation was performed using Silvaco Atlas [30]. The related material parameters for the simulation are listed in Table 1 [31,32]. The work function of the Schottky gate contact was set as the work function of the Ni metal (5.15 eV) [33]. Donor-type surface traps were set at the AlGaN/passivation layer interface with an activation energy of E_C_-0.68 eV [34] and a constant concentration of 1.2 × 10^13^ cm^−2^. These surface traps were set to compensate for the hole density on the surface [35]. The C-related traps [36] were set in the GaN buffer layer with the energy level of E_V_ + 0.9 eV as the deep acceptor trap and a constant concentration of 5 × 10^17^ cm^−3^. The buffer traps were set to compensate for the background electron density in the GaN buffer. As shown in Figure 5a, the peak electric field in the TGN-devices occurred in the region where the top gate and heterojunction interface were in contact, as depicted in the region, implying that gate leakage primarily occurred between the barrier and the top gate. However, the peak electric field in the DGN-devices occurred in the region where the sidewall gate and heterojunction interface were in contact, as depicted in region B, implying that the gate leakage primarily occurred in the sidewall depletion region. Figure 5b shows the peak electric field distribution along the nanochannel length direction. As shown in Figure 5b, with the increase in W_fin_, the peak electric field increased for both the TGN-devices and DGN-devices. For the TGN-devices, the gate leakage primarily occurred in region A, which was similar to conventional planar devices. The leakage mechanism for the TGN-devices was mainly attributed to Poole–Frenkel (PF) emission and Fowler–Nordheim (FN) tunneling and the influence of the electric field was more important. Therefore, the reverse gate leakage current of the TGN-devices increased with the increase in W_fin_. On the other hand, for the DGN-devices, the gate leakage primarily occurred in region B. During the formation of the nanochannel, the etching process could introduce etching damage and defects. The leakage mechanism for the DGN-devices was primarily associated with the sidewall-related defects, and the magnitude of leakage was jointly influenced by the electric field, trap energy levels and temperature. Moreover, the DGN-devices with smaller W_fin_ values had more sidewalls, potentially resulting in a higher total leakage current. Therefore, although the DGN-device with a W_fin_ of 150 nm presented a comparatively smaller electric field, there was still a higher total leakage current, as shown in Figure 4b.

**Figure 6:**
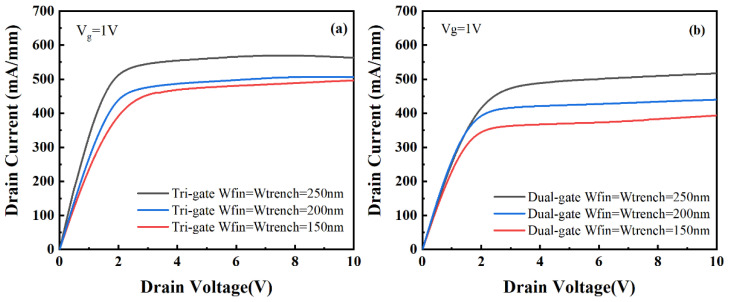
The output characteristics of the (**a**) TGN-devices and (**b**) DGN-devices.

Figure 6 shows the output characteristics of the TGN-devices and DGN-devices. As shown in Figure 6, for the same W_fin_, the TGN-devices demonstrated a higher drain current density (at gate bias of 1 V) than the DGN-devices. Moreover, for both the TGN-devices and DGN-devices, with the increase in W_fin_, the drain current density (at a gate bias of 1V) increased, which could be attributed to the increase in the overdrive voltage. Although the gate overdrive voltage (V_g_–V_th_) of the DGN-devices was higher than that of the TGN-devices for a W_fin_ of 150 nm, the saturation current of the TGN-devices was higher than that of the DGN-devices because of the higher transconductance of the TGN-devices, which was mainly attributed to the higher product of electron mobility and gate capacitance.

**Figure 7:**
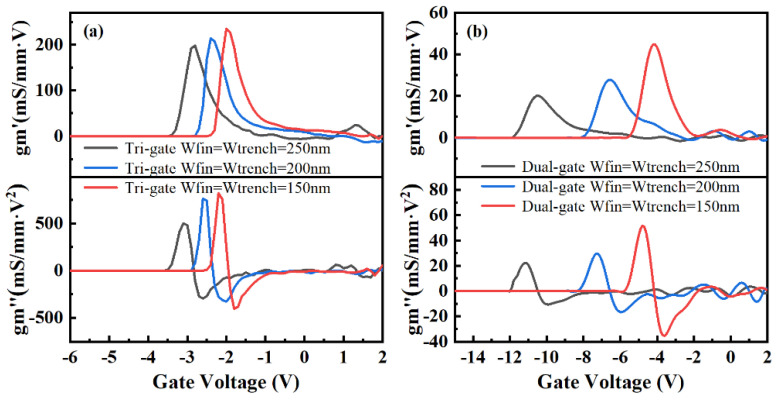
The first and second transconductance derivatives (*g*_m_′, *g*_m_″) of the (**a**) TGN-devices and (**b**) DGN-devices.

Figure 7 shows the first and second transconductance derivatives (g_m_′, g_m_″) of the TGN-devices and DGN-devices. As shown in Figure 7, for the tri-gate and dual-gate structures, as W_fin_ increased, the peak value of the first and second transconductance derivatives reduced, implying an improvement in the linearity [37]. For the same W_fin_, compared with the TGN-devices, the DGN-devices demonstrated lower first and second transconductance derivatives, indicating better linearity characteristics. It was implied that the electrostatic control from the sidewall gates was responsible for the improvement in the linearity characteristics. Moreover, the reason for the improved linearity characteristics of DGN-devices might be that the sidewall gate electrostatic control was predominant for the dual-gate structure.

**Figure 8:**
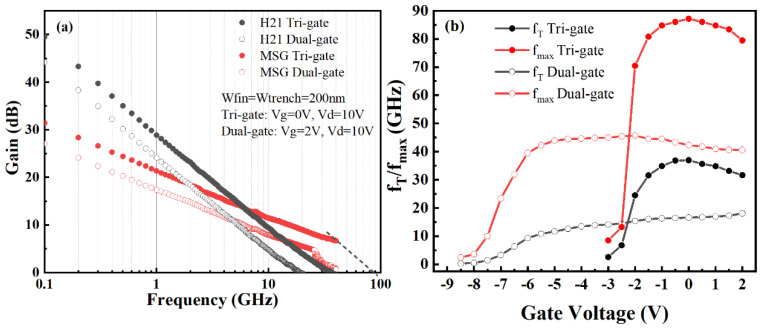
(**a**) Small-signal characteristics and (**b**) f_T_/f_max_ as a function of the gate voltage for the TGN-devices and DGN-devices. The nanochannel width was 200 nm.

To evaluate the RF characteristics of the TGN-devices and DGN-devices, the S parameters of the devices were measured using an Agilent 8363B network analyzer within the frequency range from 100 MHz to 40 GHz. The small signal characteristics of the TGN-devices and DGN-devices (W_fin_ = 200 nm) are presented in Figure 8a. f_T_ and f_max_ were extracted as the intercept of the −20 dB/decade slope for H_21_ and maximum stable gain, respectively. The f_T_/f_max_ of the TGN-devices and DGN-devices were 36.9/87.1 and 18/40.6 GHz, respectively. It can be seen that the TGN-devices had an about 51% higher f_T_ and an about 53% higher f_max_ than the DGN-devices, which was mainly attributed to the higher extrinsic transconductance of the TGN-devices. Figure 8b shows the f_T_/f_max_ dependency on the gate voltage for the TGN-devices and DGN-devices with a W_fin_ of 200 nm. It is demonstrated in Figure 8b that both the TGN-devices and DGN-devices showed a flattened f_T_/f_max_ versus gate voltage curve, which was attributed to the flattened transconductance curve profile for the TGN-devices and DGN-devices. Moreover, compared with the DGN-devices, the TGN-devices realized the higher f_T_/f_max_ but lower gate swing of f_T_/f_max_ because of the higher transconductance and the lower gate swing of transconductance for the tri-gate structure.

**Figure 9:**
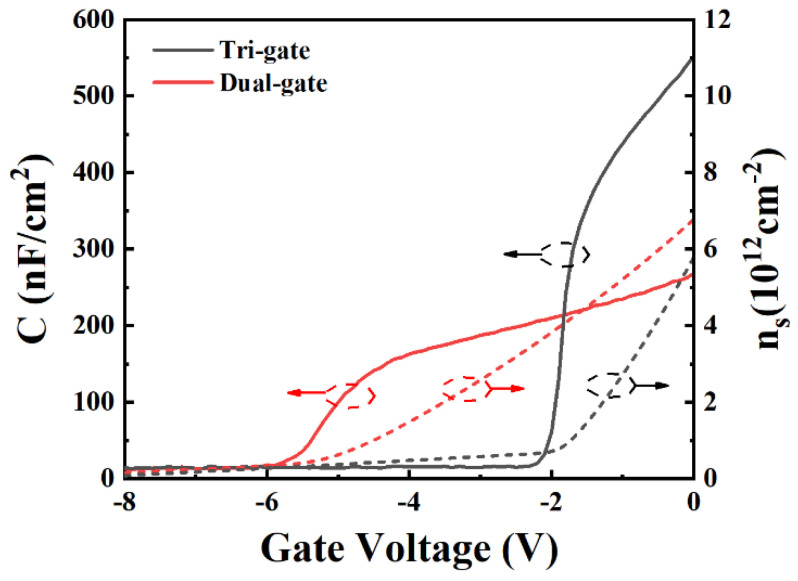
C-V curves and the electron sheet densities derived from the C-V curves for the TGN-devices and DGN-devices (W_fin_ = W_trench_ = 200 nm). The test structures were FATFETs with a gate width of 100 μm and gate length of 20 μm in order to minimize the parasitic component.

The capacitance–voltage (C-V) characteristics of the TGN-devices and DGN-devices with W_fin_ of 200 nm based on FATFET structure are shown in Figure 9. The electron sheet density (*n_s_*) is the integral of the C-V curve:

(1) $$n_{s}=\frac{1}{e}\int_{V_{pinch}}^{V_{g}}CdV$$

where *e* is the unit electron charge, *C* is the capacitance of the device, *V_g_* is the gate voltage and *V_pinch_* is the threshold voltage of the C-V curve (defined as the critical gate voltage in the C-V curve with a capacitance value less than 10 nF/cm^2^). As shown in Figure 9, the gate capacitance of the TGN-devices and DGN-devices increased when the gate voltage was larger than the threshold voltage, implying that *n_s_* nonlinearly increased with the linear increase in the gate voltage.

However, as shown in Figure 9, when the gate voltage was higher than the threshold voltage, the slope of the C-V curve for the DGN-devices was less than that of the TGN-devices, which indicated that the capacitance control of the tri-gate structure was not simply a linear superposition of top planar gate and sidewall gates (otherwise the slope of the C-V curve for both the TGN-devices and DGN-devices would be the same). Moreover, when the gate voltage was higher than the threshold voltage, the gate capacitance of the DGN-devices was lower than that of the TGN-devices, leading to the relatively slower increase in *n_s_*, which was more similar to the MESFET-like electron channel and was attributed to improving the linearity of the devices.

**Figure 10:**
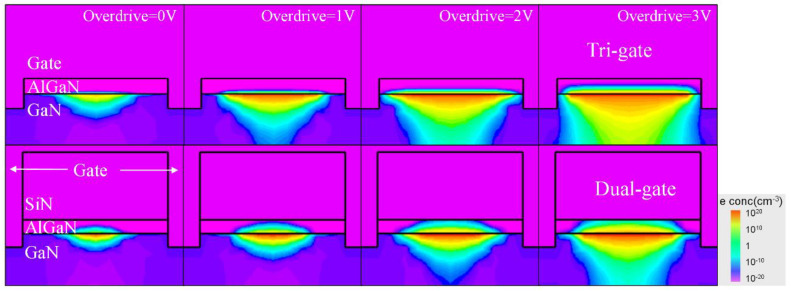
The electron concentration distribution along the gate width direction for the TGN-devices and DGN-devices with different gate overdrive voltages.

The electron concentration distributions of the TGN-devices and DGN-devices for different gate overdrive voltages are shown in Figure 10. As shown in Figure 10, the TGN-devices and DGN-devices demonstrated different gate control abilities. For the TGN-devices, applying a negative voltage to the gate electrode led to the depletion of charge in both the barrier and the channel. The top gate played a significant role in this depletion process, while the sidewall gates assisted in controlling the channel near the sidewalls. As the *V_g_* gradually became more positive, the depletion effect of the gate weakened, resulting in an increasing electron concentration in the barrier and channel. It was implied that the gate control from the top gate was dominant and the control of the electrons from the sidewall gate was assisted. For the DGN-devices, the presence of a 120 nm SiN layer between the top gate and the barrier limited the top gate’s control capability over the channel but may improve the device reliability. Therefore, the control of the electrons from the sidewall gate became primary and its control effectiveness for the central region of the barrier was weaker. As the gate overdrive voltage increased, a significant difference in the electron concentration arose between the central region of the barrier and the region near the sidewall gates. Furthermore, as shown in Figure 10, under the same gate overdrive voltage, the depletion region shape for the TGN-devices and DGN-devices were different, which was attributed to the different differential C-V curves of the tri-gate and dual-gate structures. Moreover, when the gate overdrive voltage increased, as shown in Figure 10, the *n_s_* of the TGN-devices was more than that of the DGN-devices.

## 4. Conclusions

In this study, AlGaN/GaN nanochannel HEMTs with tri-gate (TGN-devices) and dual-gate (DGN-devices) structures were fabricated and investigated. It was found that both the TGN-devices and DGN-devices demonstrated a flattened transconductance, but the G_m,peak_, f_T_ and f_max_ values of the TGN-devices were more than those of the DGN-devices because of the enhanced gate control ability from the top gate. For the same nanochannel width, the DGN-devices demonstrated lower second transconductance derivatives, implying better linearity characteristics compared with the TGN-devices. With the decrease in the nanochannel width, for both the TGN-devices and DGN-devices, the peak value of transconductance and the first and second transconductance derivatives increased, implying the predominant influence of sidewall gate capacitance on the transconductance and linearity. It was demonstrated that the gate capacitance of the tri-gate structure was not simply a linear superposition of the top planar gate capacitance and sidewall gate capacitance of the dual-gate structure, which could be attributed to the difference in the depletion region shape for the tri-gate and dual-gate structures.

## Figures and Tables

**Table 1:** Material parameters (bandgap, electron effective masses in the growth direction and perpendicular to the growth direction, polarization constants and lattice constants) used for the simulations [31,32]. The description of Table 1 gives the explanation of the simulation setup parameters. m║ * and m┴ * stand for electron effective masses in the growth direction and perpendicular to the growth direction, respectively.

	GaN	AlN	Al_0.23_Ga_0.77_N
E_g_ (300 K) (eV)	3.42	6.28	4.08
m║ *	0.18	0.25	0.20
m┴ *	0.20	0.33	0.23
e_33_ (C/m^2^)	0.73	1.46	0.90
e_31_ (C/m^2^)	−0.49	−0.60	−0.51
a_0_ (Å)	3.189	3.112	3.171
c_0_ (Å)	5.185	4.982	5.138
